# Evaluating Growth and Stability of Nine Poplar Clones for Riparian Afforestation

**DOI:** 10.3390/plants14162482

**Published:** 2025-08-10

**Authors:** Jihyeon Jeon, Hyemin Lim, Kyungmi Lee, Eun Woon Noh, Il Hwan Lee, Wi Young Lee, Yeong Bon Koo, Kyunghwan Jang

**Affiliations:** 1 Department of Forest Bioresources, National Institute of Forest Science, Suwon 16631, Republic of Korea; w920827@gmail.com (J.J.); kmile@korea.kr (K.L.); lih0618@korea.kr (I.H.L.); 2 Korea National Commission on Poplars and Other Fast-Growing Trees, Suwon 16631, Republic of Korea; ewnoh1@naver.com (E.W.N.); wylee20@naver.com (W.Y.L.); ybkoo1242@naver.com (Y.B.K.); jkh67360@naver.com (K.J.)

**Keywords:** *Populus*, survival, growth performance, clone selection, site adaptability

## Abstract

Poplar (*Populus*) clones are widely used for riparian afforestation owing to their fast growth and ecological benefits. However, selecting suitable clones for site-specific conditions remains a key challenge. In this study, we evaluated the survival and growth performance of nine poplar clones belonging to three hybrid groups—*Populus deltoides* (D), *P. deltoides* × *P. nigra* (DN), and *P. nigra* × *P. suaveolens* (NS)—at two riparian sites in Korea. Significant differences were observed in the survival, height, diameter, basal area, and basal area increment (BAI) among clones and between sites. DN hybrids exhibited superior overall performance in both survival and growth traits compared to D and NS clones. In the DN group, clones Eco-28, I-476, and Dorskamp consistently ranked highest in aggregate performance. Notably, I-476 and Eco-28 demonstrated both high productivity and stability across sites, as reflected in their low coefficients of variation (CVs). In contrast, Dorskamp, while highly productive, showed relatively high variability across environments. These findings highlight DN hybrids—particularly Eco-28 and I-476—as promising candidates for riparian afforestation, offering a balanced combination of high productivity and environmental stability.

## 1. Introduction

In Korea, where approximately two-thirds of the land is mountainous, flat areas suitable for large-scale tree plantations are limited [1]. Among the few available options, riparian zones along major rivers, such as the Han River—the largest river in Korea—have gained increasing attention for afforestation and land-use management. In recent years, recreational activities such as cycling, running, and walking have become relatively popular along the Han River, highlighting the growing multifunctional role of riverside forests [2]. These forests serve various public purposes beyond timber production, including landscape enhancement, air quality improvement, shade provision, noise reduction, and urban heat island mitigation [3]. Managed primarily by local governments, urban riparian forests are often integrated into broader urban and regional planning strategies.

Poplar (*Populus*) trees are particularly well suited for riparian afforestation owing to their rapid growth, adaptability, and ecological functions. Their fast canopy development enhances shade and recreational value [4], while their deciduous nature allows for seasonal light penetration, supporting biodiversity [5]. Poplars also function as effective riparian buffers by reducing nutrient runoff, filtering pollutants such as nitrogen and phosphorus before they enter waterways [6]. In addition to their ecological benefits, poplars contribute to biomass production; for example, Fortier et al. [7] reported yields exceeding 100 t ha^−1^ of dry matter after 6 years in riparian plantations. Moreover, poplars and willows are widely used for applications such as phytoremediation and floodplain restoration [8]. Despite these advantages, potential concerns such as higher water consumption [9,10] and invasiveness [11,12] must be carefully considered when selecting species for riparian afforestation.

Although poplars exhibit broad adaptability, growth performance varies substantially, depending on species, genotype, and environmental conditions [13]. Hybridization offers the potential to combine desirable traits from parental species, enhancing growth rates and site adaptability [14,15,16]. Hybrid poplars have been widely used for their superior growth and flexibility, yet their survival and productivity are still strongly influenced by site-specific factors [17]. Among these, soil nutrient availability has been identified as a critical factor for successful poplar establishment and growth [18,19].

While *Populus deltoides* and *P. nigra* are not native to Korea, they were introduced in the early 20th century and have been widely planted nationwide since the 1950s [20]. Among them, poplar clones and hybrids—particularly *Populus deltoides* (native to North America) and *P. nigra* (native to Europe and Southwest Asia)—have been extensively used in afforestation and landscaping projects [21,22,23]. In contrast, only *P. suaveolens* is considered native to Korea [24]. Given the widespread use of these alien species and their hybrids, it is essential to assess their potential ecological impacts. The introduction of non-native poplars into riparian zones may affect native plant communities through mechanisms such as competition, hybridization, and alteration of habitat structure and ecosystem functions [25,26,27].

Despite their promising potential, studies assessing the performance of different poplar clones under real-world riparian conditions in Korea are relatively few. Most existing research has focused on upland environments or controlled experimental settings, with limited understanding of how genetic variation interacts with site-specific factors such as soil fertility, water availability, and urban microclimates along rivers. Moreover, little attention has been paid to evaluating both productivity and stability—key traits for ensuring long-term ecosystem services in multifunctional riparian forests. To guide evidence-based clone selection and promote successful riparian afforestation, conducting multi-trait assessments across representative riparian environments is essential.

This study aims to address these gaps through evaluation of the performance of nine poplar clones at two urban riparian sites in Korea. The specific objectives of the study are as follows: (1) to assess the survival and growth characteristics of each clone under riparian site conditions; (2) to identify clones that exhibit both high productivity and environmental stability, thereby supporting their suitability for riparian afforestation programs.

## 2. Materials and Methods

### 2.1. Plant Materials

Nine poplar clones were used in this study (Table 1): three *Populus deltoides* × *P. deltoides* (D) crosses, four *P. deltoides* × *Populus nigra* (DN) hybrid clones, and two *P. nigra* × *Populus suaveolens* (NS) hybrid clones. These superior clones were selected from hundreds of candidates evaluated over 50 years of field trials at the National Institute of Forest Science (NIFoS), based on biomass production and phytoremediation potential in degraded land [28].

In spring 2016, approximately 20 cm cuttings of the selected clones were harvested from current-year shoots in stool beds at NIFoS. The cuttings were then directly planted in the nursery. The rooted cuttings were harvested as bare-root seedlings in 2017 and subsequently transferred and planted at the designated planting sites.

### 2.2. Study Sites

**Figure 1:**
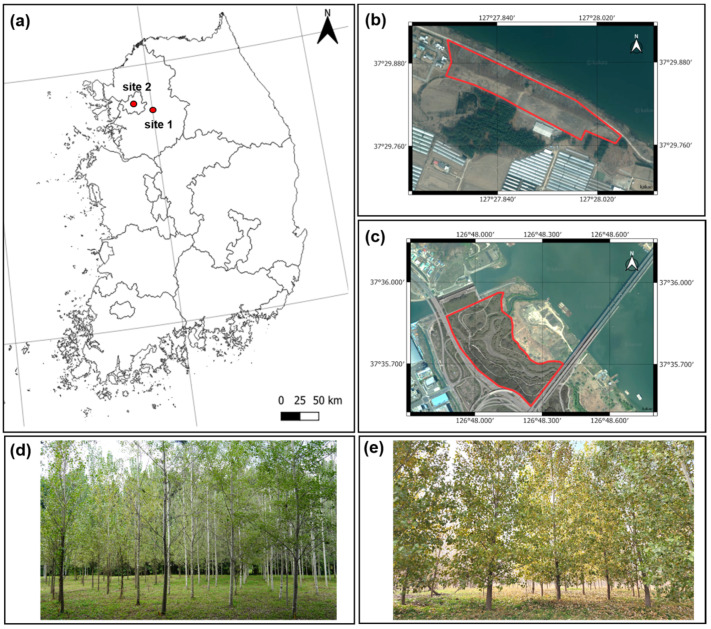
(**a**) Locations of the two riparian study sites in Korea (red dots). (**b**,**d**) Site 1 (Yangpyeong). (**c**,**e**) Site 2 (Seoul). The red-lined area is the subject of the study.

Study sites were established at two riparian locations in the Republic of Korea (Figure 1). Site 1 (Yangpyeong) was a former wood plantation located in Gyeonggi Province (N 37°29′48″ E 127°27′57″; 27 m a.s.l.). It is situated between a paddy field and the river, approximately 15 m above the river surface. Site 2 (Seoul) was a former reed field located in Seoul, the capital city of Korea (N 37°35′40″ E 126°48′01″; 6 m a.s.l.). It is a floodplain between the Han River and a heavily trafficked highway and is situated less than 5 m above the river surface. 

Climatic data for the study period (2017 to 2024) were obtained from the Open Met Data Portal of the National Climate Data Center (https://data.kma.go.kr/cmmn/main.do, accessed on 4 March 2025). During this period, annual precipitation was similar between the two study sites (Site 1: 1375 ± 357 mm; Site 2: 1366 ± 290 mm). However, the mean annual temperature at Site 2 (13.6 ± 0.7 °C) was 0.9 °C higher than that at Site 1 (12.7 ± 0.6 °C).

### 2.3. Experimental Setup

To account for within- and between-site variability, a randomized complete block design with three replicate blocks was established at each site. Each block contained nine rows, with each row representing a different species or hybrid. In total, 17 trees were planted per row at Site 1 and 20 trees were planted per row at Site 2. Planting density was 800 trees/ha (5 × 2.5 m) at Site 1 and 625 trees/ha (4 × 4 m) at Site 2. Although planting density may influence tree growth, its effect was considered negligible in this study due to the relatively small difference between the two sites.

Planting was carried out in April 2017. Prior to planting, both sites were mechanically weeded. Weeding was conducted twice annually (in May and August) until the second year. As both sites were located near the river, to prevent potential water pollution, no fertilizer was applied.

### 2.4. Survival and Grwoth Data

Growth (height and diameter at breast height) and survival data for each block at the study sites were collected in May 2024. Tree height was measured using a fishing rod marked in centimeters, and the diameter at breast height (DBH) was measured at 1.2 m above ground level using a diameter tape for all individuals within each replicate block. The basal area (BA, cm^2^) was calculated for each tree using the following formula:

$$BA=\frac{\pi \times {DBH}^{2}}{4}$$

where the DBH is measured in centimeters. The total basal area per hectare was obtained by summing the BAs of all surviving trees within each block and scaling by the planting density. The basal area increment (BAI) was estimated as the mean annual basal area growth, calculated by dividing the basal area at year 7 by the stand age (7 years).

### 2.5. Soil Sampling and Analysis

The topsoil depth was determined by digging a hole approximately 50 cm in diameter and 40–50 cm deep at the center of each plot. The maximum depth of root penetration was regarded as the effective soil depth. Both sites had flat topography, but the topsoil was deeper at Site 1 (up to <60 cm) compared to at Site 2 (approximately 40 cm). For analyses of soil texture and chemical properties, approximately 500 g of soil was collected from a depth of 5–10 cm below the organic layer in each plot. This depth was chosen as it represents the overall soil conditions, given the lack of stratification within the effective soil depth. The samples were air-dried indoors and passed through a 2 mm sieve prior to analysis. All soil analyses were performed by the Korea Forestry Promotion Institute (https://ksfer.or.kr/service, accessed on 29 November 2024). To determine soil texture, particle size distribution was analyzed using the hydrometer method [29], and soil texture was classified according to the United States Department of Agriculture (USDA) classification system. For chemical property analyses, the following parameters were measured: soil pH, organic matter, available phosphorus (P_2_O_5_), silicon oxide (SiO_2_), sodium ion (Na^+^), NaCl, and exchangeable cations (K^+^, Ca^2+^, and Mg^2+^). Soil pH was measured in a 1:2.5 soil-to-water suspension using a pH meter (HM-30R, DKK-TOA Corporation, Tokyo, Japan) [30]. Organic matter and total nitrogen contents were analyzed using the dry combustion method (Dumas method) with an elemental analyzer (US/Vario Max CN, Elementar Analysensysteme GmbH, Langenselbold, German) [31]. Available phosphorus was determined using the Lancaster method with a UV–visible spectrophotometer (Cary 4000, Varian Inc., Palo Alto, CA, USA) [32]. Cation exchange capacity was measured using 1N-ammonium acetate extraction [33]. Exchangeable cations were analyzed by atomic absorption spectrophotometry using an atomic absorption spectrometer (AA 280FS, Varian Inc., Palo Alto, CA, USA) [34].

### 2.6. Statistical Analyses

All statistical analyses were conducted using R software (version 4.4.3) [35]. Descriptive statistics (mean ± standard deviation) were calculated for each clone and site. Data normality was tested using the Shapiro–Wilk test, and homogeneity of variance was assessed using Levene’s test. The majority of variables showed normal distribution (*p* > 0.05) and satisfied the assumption of homogeneity. For variables that violated these assumptions—such as phosphorus pentoxide, exchangeable Ca, and ion conductivity (*p* < 0.05)—appropriate alternative statistical tests (e.g., Welch’s *t*-test or Welch’s ANOVA) were applied. Differences in soil chemical properties between sites were evaluated using independent *t*-tests. For variables with unequal variances, Welch’s *t*-tests were employed, whereas Student’s *t*-tests were used for the remaining variables. For survival rate, differences between sites were analyzed using Welch’s *t*-test, while differences among clones and the clone × site interaction were assessed using two-way ANOVA. For height and diameter, differences between sites were analyzed using Student’s *t*-test, while differences among clones and the clone × site interaction were assessed using Welch’s ANOVA. An aggregate ranking was calculated for each clone by summing ranks for survival, DBH, volume, and BAI, with relatively low total ranks indicating superior overall performance. Clonal stability was assessed by calculating the coefficient of variation (CV%) for each trait, where lower CV values indicate greater stability across sites. Spearman’s rank correlation was used to examine consistency between traits. Statistical significance was determined at *p* < 0.05.

## 3. Results

### 3.1. Soil Texture and Chemical Properties

The soil texture at both Site 1 (Yangpyeong) and Site 2 (Seoul) was classified as silt loam (Table 2). There were no significant differences between the two sites in soil pH, organic matter content, phosphorus pentoxide, silicic acid, and ion conductivity (*p* > 0.05). In contrast, several chemical properties showed significant differences. Site 2 had higher concentrations of exchangeable potassium (0.46 ± 0.10 cmol/kg), calcium (6.17 ± 1.10 cmol/kg), and magnesium (1.20 ± 0.17 cmol/kg) than Site 1 (0.13 ± 0.10, 0.33 ± 0.21, and 0.13 ± 0.06 cmol/kg, respectively; *p* = 0.014, 0.010, and <0.001). Additionally, sodium exchangeable percentage (ESP%) was significantly higher at Site 2 (0.26 ± 0.04%) than at Site 1 (0.03 ± 0.01%, *p* < 0.001).

### 3.2. Survival Rate

The survival after the 7th growing season is shown in Table 3. Significant differences in survival rates were observed between sites (*p* < 0.01), among clones (*p* < 0.01), and for the clone × site interaction (*p* < 0.01). The mean survival rates of the clones were 60.1 ± 28.7% at Site 1 and 82.8 ± 13.5% at Site 2. At both sites, the DN hybrids had the highest survival rate (82.7 ± 18.4%), followed by the NS clones (75.8 ± 16.0%) and D clones (53.6 ± 28.3%). Among the D clones, the 97-19 clone (*P. deltoides* (Lux) × *P. deltoides* (Havard)) had the lowest survival rate at both sites. In contrast, the DN hybrids consistently showed the highest survival, with the I-476 clone (*P. deltoides* × *P. nigra*) demonstrating particularly strong performance at both locations.

### 3.3. Growth Performance

**Figure 2:**
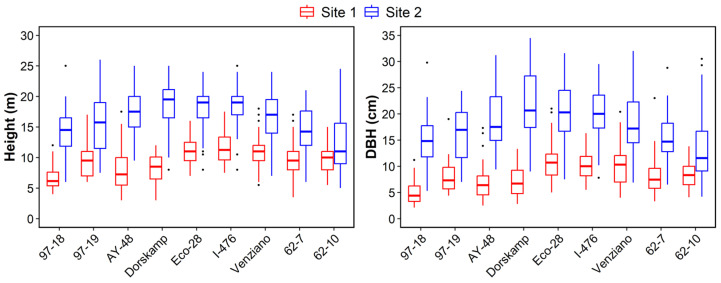
Height (**Left**) and breast height diameter (**Right**) growth of *Populus* clones in two riparian sites (Site 1: Yangpyeong, Site 2: Seoul). The box size represents the interquartile range; the horizontal solid black line within the boxes is the median, and the whiskers indicate variability outside the upper and lower quartiles. Dots outside the whiskers represent outliers.

Height and diameter growth data were obtained from measurements of the surviving trees. Figure 2 shows boxplots of tree height and diameter at breast height (DBH). Significant differences were observed in both height and diameter growth between sites (*p* < 0.01) and among clones (*p* < 0.01). In both traits, the growth performance of clones at Site 1 (red boxes) was consistently lower than that at Site 2 (blue boxes) (*p* < 0.001). The mean height growth of the clones was 10.0 ± 2.9 m at Site 1 and 16.5 ± 4.4 m at Site 2. The mean DBH was 8.9 ± 3.6 cm at Site 1 and 18.0 ± 6.1 cm at Site 2.

The DN clones exhibited significantly greater height and diameter growth than both the D and NS clones (*p* < 0.05). At Site 1, the DN clones showed the greatest growth in both height and diameter, while the D clones had the lowest values (*p* < 0.01). At Site 2, the DN clones again showed the highest growth, whereas the NS clones showed the lowest growth (*p* < 0.001).

At Site 1, the top three clones for height were DN I-476 (11.6 ± 2.3 m), DN Eco-28 (11.0 ± 2.1 m), and DN Venziano (10.7 ± 2.8 m) at Site 1. For DBH, the leading clones were DN Eco-28 (10.9 ± 3.6 cm), DN I-476 (10.1 ± 2.7 cm), and DN Venziano (10.0 ± 3.9 cm). At Site 2, the tallest clones were DN Dorskamp (18.8 ± 3.7 m), DN I-476 (18.4 ± 2.7 m), and DN Eco-28 (18.0 ± 4.0 m), while the clones with the greatest diameter growth were DN Dorskamp (22.0 ± 6.3 cm), DN I-476 (20.3 ± 4.8 cm), and DN Eco-28 (20.0 ± 5.9 cm) at Site 2. All values are presented as mean ± SD.

**Figure 3:**
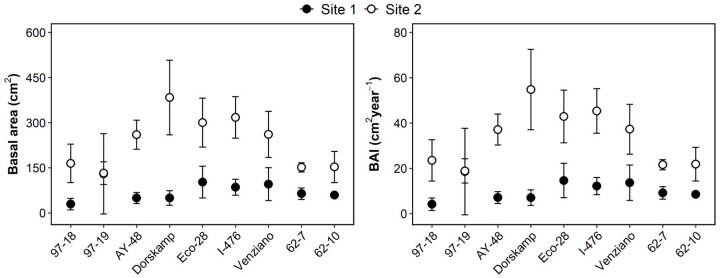
Comparison of the basal area (**left**) and the basal area increment (**right**) among clones grown at Site 1 (closed circles) and Site 2 (open circles). Data are presented as means ± standard deviations.

The basal area and basal area increment (BAI) varied considerably among clones and between sites (Figure 3). Overall, clones grown at Site 2 exhibited a substantially higher basal area and BAI than those at Site 1. At Site 2, Dorskamp recorded the highest mean basal area, exceeding 400 cm^2^, followed by clones I-476 and Eco-28. In contrast, basal area values at Site 1 remained consistently lower across all clones, generally below 150 cm^2^. A similar pattern was observed for the BAI, with Dorskamp exhibiting the highest increment (~60 cm^2^ year^−1^) at Site 2, while BAI values at Site 1 were markedly lower, mostly below 20 cm^2^ year^−1^.

### 3.4. Consistency Between Survival and Growth Performance and Stability Evaluation

**Figure 4:**
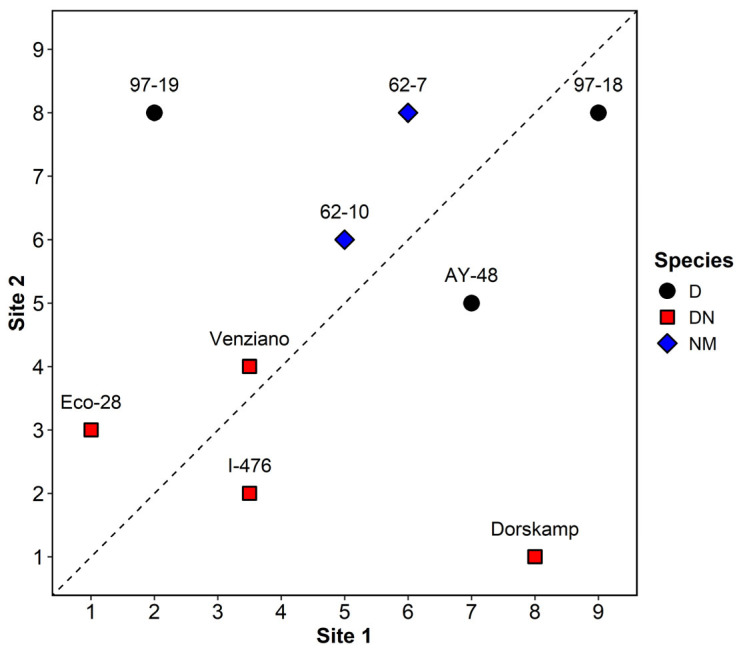
Comparison of total clone performance ranks between Site 1 and Site 2. Lower ranks indicate better performance. The dotted line represents a 1:1 correspondence. Clones near the line performed consistently across sites.

Total clone performance ranks varied notably between the two sites (Figure 4). DN hybrids such as Dorskamp, I-476, and Eco-28 consistently ranked high (i.e., low rank values) at both sites and were positioned near the 1:1 line, suggesting stable performance across environments. In contrast, D clones including 97-18 and 97-19 exhibited large rank discrepancies, performing poorly at Site 1 but ranking much higher at Site 2. NS hybrids (62-7, 62-10) showed moderate to inconsistent shifts in rank between sites. Spearman’s rank correlation between Site 1 and Site 2 was r = 0.14, *p* = 0.727, indicating only weak and statistically non-significant agreement in overall clone performance across the two environments.

An aggregate ranking approach was applied to evaluate overall clone performance by combining survival, DBH, volume, and BAI (Table 4). The aggregate ranks revealed substantial variation among clones. Clones belonging to the *Populus deltoides* × *P. nigra* (DN) hybrid group generally exhibited superior overall performance compared to the other taxonomic groups. In particular, Eco-28, I-476, and Dorskamp consistently ranked highest across all evaluated traits, indicating strong adaptability and suitability for riparian afforestation and biomass production under the studied conditions.

To further assess clonal stability, the coefficient of variation (CV%) was calculated for survival and growth traits (Table 4). Lower CV values reflect relatively high consistency across varying environmental conditions. Among DN hybrids, I-476 and Eco-28 demonstrated the lowest CVs for survival (6.2% and 9.2%, respectively), as well as relatively low variation in DBH, volume, and BAI. These results indicated that I-476 and Eco-28 exhibited high growth performance and maintained stable outcomes across sites. Although Dorskamp showed excellent mean growth values, its relatively high CVs for volume (108.2%) and BAI (92.2%) suggest relatively high sensitivity to site-specific variability. Overall, I-476 and Eco-28 emerged as the most favorable clones when both productivity and stability are considered for riparian afforestation efforts.

## 4. Discussion

### 4.1. Clonal Differences and Superiority of DN Hybrids in Riparian Conditions

This study revealed substantial clonal variation in survival and growth performance among nine poplar clones planted in two urban riparian sites in Korea (Table 3, Figure 2). Among the three parentage groups, DN hybrids (*Populus deltoides* × *P. nigra*) consistently outperformed D clones (*P. deltoides*) and NS hybrids (*P. nigra* × *P. suaveolens*) across all measured traits, including height, DBH, basal area, and BAI. In particular, Eco-28, I-476, and Dorskamp achieved high growth metrics (Figure 3) and survival rates (Table 3), demonstrating superior adaptability to riparian conditions. These results corroborate earlier findings highlighting the hybrid vigor of DN clones, which often show enhanced biomass accumulation and environmental tolerance [36,37,38]. I-476 was especially notable for maintaining the highest and most stable survival across both sites, indicating its potential as a broadly adaptable candidate for riparian afforestation. The superior performance of DN hybrids may stem from favorable traits such as fast canopy development, deeper root systems, and moderate water use efficiency—characteristics that are particularly beneficial in periodically moist but nutrient-variable riparian environments.

### 4.2. Environmental Drivers of Site-Specific Performance Variation

Despite the superior overall performance of DN hybrids, clone rankings differed considerably between the two sites (Figure 4), and Spearman’s rank correlation for total trait performance was weak and non-significant (r = 0.14, *p* = 0.727), suggesting strong site effects. The survival at Site 1 (60.1%) was significantly lower than that at Site 2 (82.8%), and this divergence was accompanied by lower DBH, volume, and BAI at Site 1 (Figure 3). Soil analysis (Table 2) indicated that Site 2 had higher levels of exchangeable Ca, Mg, and ESP%, while Site 1 showed signs of nutrient depletion and low pH, potentially driven by past land-use practices such as repeated tree cultivation and harvesting. The nutrient-poor conditions and possible leaching at Site 1 may have disproportionately impacted D clones such as 97-18 and 97-19, which showed poor survival and growth there but performed better at Site 2. This result suggests that these clones may possess physiological traits that are less tolerant to acidic or nutrient-deficient conditions, such as higher stomatal sensitivity or shallow rooting depth [39,40]. These observations support the need for multi-site trials and highlight the role of local edaphic factors in clone adaptability and afforestation success.

### 4.3. Integrative Evaluation of Clonal Productivity and Stability

To identify clones that combine high productivity with inter-site stability, we employed an aggregate ranking approach across survival, DBH, volume, and BAI (Table 4). Eco-28 and I-476 consistently ranked in the top tier and exhibited a low coefficient of variation (CV%) for most traits, including survival (9.2% and 6.2%, respectively), indicating reliable performance across different site conditions. In contrast, Dorskamp showed excellent growth at Site 2 but relatively high CVs for volume (108.2%) and BAI (92.2%), suggesting relatively high sensitivity to environmental variability. These results align with those of prior studies on genotype × environment (G × E) interactions in hybrid poplars [17,41] and highlight the importance of stability metrics, in addition to absolute trait values. Notably, the contrasting survival rates between sites suggest that differences in basal area and productivity were not merely the result of density-dependent effects (e.g., self-thinning) but were likely driven by differences in soil nutrient status. Therefore, clone selection should consider both mean performance and response variability across site types.

### 4.4. Implications for Clone Selection and Future Research in Riparian Forestry

This study provides practical insights into clone selection for riparian afforestation under urban and peri-urban conditions. DN hybrids—particularly Eco-28 and I-476—stand out as superior options, offering both high productivity and environmental consistency. Their successful deployment could help achieve multifunctional riparian objectives, including biomass production, shade provision, pollution mitigation, and aesthetic enhancement in Korea’s constrained lowland areas. The integrated use of performance ranking and CV-based stability assessment offers a replicable framework for clone evaluation in future afforestation planning. While these clones demonstrated strong adaptability, it is also important to consider the ecological implications of introducing alien genetic material—such as *Populus deltoides* and *P. nigra*—into sensitive riparian ecosystems [7,42,43]. Potential impacts on native biodiversity and ecosystem function should be assessed in parallel with productivity. Continued studies are needed to elucidate the underlying physiological or anatomical traits that contribute to clone-specific adaptability—particularly for less stable clones such as Dorskamp or the more sensitive D group. Future research should incorporate long-term monitoring of eco-physiological responses, such as water use strategies, root architecture, and stress tolerance under fluctuating hydrological regimes. Moreover, expanding trials to additional sites with varying soil fertility and hydrology will further refine clone recommendation systems and enhance the success of riparian forest establishment across different landscapes. In addition, given the widespread and long-term use of exotic poplar species in Korea, future research should also include ecological assessments of their potential interactions with native riparian ecosystems.

## 5. Conclusions

In this study, the survival, growth performance, and stability of nine *Populus* clones under riparian conditions were evaluated across two urban sites in Korea. DN hybrids (*Populus deltoides* × *P. nigra*), particularly Eco-28, I-476, and Dorskamp, exhibited superior growth and survival performance. Among these, I-476 and Eco-28 demonstrated high stability across sites, suggesting strong adaptability to varying environmental conditions. These results highlight the importance of considering both productivity and stability in clone selection for afforestation programs. Our findings indicate that I-476 and Eco-28 are promising candidates for riparian afforestation, particularly in heterogeneous environments where consistent stand development is essential. Dorskamp, despite its high productivity, exhibited relatively considerable variability and may be relatively suitable for site-specific applications under favorable conditions. This study contributes practical insights for selecting *Populus* clones that balance growth potential with ecological resilience, supporting the development of productive and stable riparian plantations. Considering the ecological importance and environmental sensitivity of riparian zones, careful clone selection is critical for ensuring long-term afforestation success. Future research should conduct longer-term monitoring of stand dynamics and evaluate additional factors such as resistance to biotic and abiotic stressors, ecosystem service impacts, and the potential for natural regeneration. These efforts will further elucidate clone adaptability and guide sustainable management practices under changing climatic and site conditions.

## Figures and Tables

**Table 1:** Poplar clones and their parentages used in the study.

Pedigree	Group	Clone
*P. deltoides* × *P. deltoides*	D	97-18, 97-19, and AY48
*P. deltoides* × *P. nigra*	DN	Dorskamp, Eco28, I-476, and Venziano
*P. nigra* × *P. suaveolens*	NS	62-7 and 62-10

**Table 2:** Soil texture and chemical properties at the study sites (mean ± SD, n = 3).

	Site 1 (Yangpyeong)	Site 2 (Seoul)	*p*-Value
Soil texture	Silt loam	Silt loam	
Soil pH	5.2 ± 0.2	6.0 ± 0.6	0.081
Organic matter (g/kg)	16.3 ± 8.5	18.7 ± 10.1	0.775
Phosphorus pentoxide (mg/kg)	65.0 ± 7.5	80.0 ± 65.3	0.730
Exchangeable K (cmol/kg)	0.13 ± 0.10	0.46 ± 0.10	0.014
Exchangeable Ca (cmol/kg)	0.33 ± 0.21	6.17 ± 1.10	0.010
Exchangeable Mg (cmol/kg)	0.13 ± 0.06	1.20 ± 0.17	<0.001
Silicic acid (mg/kg)	91.0 ± 12.1	155.0 ± 41.3	0.062
Ion conductivity (dS/m)	0.13 ± 0.06	0.33 ± 0.23	0.270
Na (ESP%)	0.03 ± 0.01	0.26 ± 0.04	<0.001

**Table 3:** Seven-year survival performance of poplar clones at the study sites (mean ± SD).

Group	Clone	Survival Rate (%)	
		Site 1	Site 2
D	97-18	23.5 ± 15.6	80.0 ± 10.0
	97-19	17.6 ± 15.6	56.7 ± 5.8
	AY-48	58.8 ± 15.6	85.0 ± 8.7
DN	Dorskamp	45.1 ± 6.8	93.3 ± 2.9
	Eco-28	84.3 ± 9.0	88.3 ± 7.6
	I-476	98.0 ± 3.4	93.3 ± 7.6
	Venziano	70.6 ± 21.2	88.3 ± 5.8
NS	62-7	62.7 ± 14.8	73.3 ± 12.6
	62-10	80.4 ± 18.0	86.7 ± 15.3

**Table 4:** Clonal stability (CV%) and aggregate ranking for the survival, DBH, volume, and BAI.

Group	Clone	CV				Rank
		Survival	DBH	Volume	BAI	
D	97-18	64.1	53.2	107.7	88.3	9
	97-19	63.9	36.7	68.1	67.0	6
	AY-48	25.4	51.8	96.1	77.3	5
DN	Dorskamp	38.9	56.8	108.2	92.2	3
	Eco-28	9.2	38.1	89.8	61.9	1
	I-476	6.2	39.5	85.5	67.3	2
	Venziano	21.4	37.5	84.2	60.9	4
NS	62-7	19.9	34.7	70.7	46.8	8
	62-10	18.3	34.7	105.5	57.4	7

## Data Availability

Data are contained within the article.

## References

[1] Rahman G., Kim J.Y., Kim T.W., Park M., Kwon H.H. (2025). Spatial and Temporal Variations in Temperature and Precipitation Trends in South Korea over the Past Half-Century (1974–2023) Using Innovative Trend Analysis. J. Hydro-Environ. Res..

[2] Yun J. (2022). The Han River Development: Planning the Riverfront as Seoul’s Natural Landmark. Sustainability.

[3] Naiman R.J., Fetherston K.L., McKay S.J., Chen J., Naiman R.J., Bilby R.E. (1998). Riparian Forests. River Ecology and Management: Lessons from the Pacific Coastal Ecoregion.

[4] Johnson M.F., Wilby R.L. (2015). Seeing the Landscape for the Trees: Metrics to Guide Riparian Shade Management in River Catchments. Water Resour. Res..

[5] Zellweger F., Coomes D., Lenoir J., Depauw L., Maes S.L., Wulf M., De Frenne P. (2019). Seasonal Drivers of Understorey Temperature Buffering in Temperate Deciduous Forests across Europe. Glob. Ecol. Biogeogr..

[6] Bahn G.-S., An B.-C. (2020). Analysis of Environmental Purification Effect of Riparian Forest with Poplar Trees for Ecological Watershed Management: A Case Study in the Floodplain of the Dam Reservoir in Korea. Sustainability.

[7] Fortier J., Truax B., Gagnon D., Lambert F. (2016). Potential for Hybrid Poplar Riparian Buffers to Provide Ecosystem Services in Three Watersheds with Contrasting Agricultural Land Use. Forests.

[8] Isebrands J.G., Richardson J. (2014). Poplars and Willows: Trees for Society and the Environment.

[9] Théroux Rancourt G., Éthier G., Pepin S. (2015). Greater Efficiency of Water Use in Poplar Clones Having a Delayed Response of Mesophyll Conductance to Drought. Tree Physiol..

[10] Xi B., Clothier B., Coleman M., Duan J., Hu W., Li D., Fernández J.E. (2021). Irrigation Management in Poplar (*Populus* spp.) Plantations: A Review. For. Ecol. Manag..

[11] Sikorska D., Sikorski P., Archiciński P., Chormański J., Hopkins R.J. (2019). You Can’t See the Woods for the Trees: Invasive Acer negundo L. in Urban Riparian Forests Harms Biodiversity and Limits Recreation Activity. Sustainability.

[12] Havrdová A., Douda J., Doudová J. (2023). Threats, Biodiversity Drivers and Restoration in Temperate Floodplain Forests Related to Spatial Scales. Sci. Total Environ..

[13] Richardson J., Isebrands J.G., Ball J.B., Isebrands J.G., Richardson J. (2014). Ecology and Physiology of Poplars and Willows. Poplars and Willows: Trees for Society and the Environment.

[14] Tsarev A., von Wühlisch G., Tsareva R. (2017). Hybridization of Poplars in the Central Chernoff-Zem Region of Russia. Silvae Genet..

[15] Boothroyd-Roberts K., Gagnon D., Truax B. (2013). Can Hybrid Poplar Plantations Accelerate the Restoration of Forest Understory Attributes on Abandoned Fields?. For. Ecol. Manag..

[16] Qin G.-H., Jiang Y.-Z., Qiao Y.-L. (2013). Selection of Poplar Hybrid Clones (*Populus* spp.) from Backcrossed Progenies of the Aigeros Section for Industrial Purpose. Silvae Genet..

[17] Nelson N., Meilan R., Berguson W., McMahon B., Cai M., Buchman D. (2019). Growth Performance of Hybrid Poplar Clones on Two Agricultural Sites with and without Early Irrigation and Fertilization. Silvae Genet..

[18] Yu C.-B., Chen F., Luo Z.-J. (2004). Evaluation of Soil Nutrient Status in Poplar Forest Soil by Soil Nutrient Systematic Approach. J. For. Res..

[19] Ghezehei S.B., Ewald A.L., Hazel D.W., Zalesny R.S., Nichols E.G. (2021). Productivity and Profitability of Poplars on Fertile and Marginal Sandy Soils under Different Density and Fertilization Treatments. Forests.

[20] Kim K.H., Zsuffa L. (1994). Reforestation of South Korea: The history and analysis of a unique case in forest tree improvement and forestry. For. Chron..

[21] Rajora O.P., Zsuffa L. (1991). Screening *Populus deltoides* Marsh. selections by allozymes to assure species identity. Scand. J. For. Res..

[22] Stobrawa K. (2014). Poplars (*Populus* spp.): Ecological role, applications and scientific perspectives in the 21st century. Balt. For..

[23] De Rigo D., Enescu C.M., Houston Durrant T., Caudullo G. (2016). Populus nigra in Europe: Distribution, habitat, usage and threats. European Atlas of Forest Tree Species.

[24] Kim C.W., Cha D.S., Choi Y.E., Koo Y.B., Choi W.Y., Oh J.H., Yi J.S. (2009). Researches on *Populus* in Korea for various purposes. J. Korean For. Soc..

[25] Castro-Díez P., Alonso Á. (2017). Effects of non-native riparian plants in riparian and fluvial ecosystems: A review for the Iberian Peninsula. Limnetica.

[26] Meyer S.E., Callaham M.A., Stewart J.E., Warren S.D. (2021). Invasive species response to natural and anthropogenic disturbance. Invasive Species in Forests and Rangelands of the United States: A Comprehensive Science Synthesis for the United States Forest Sector.

[27] Gazoulis I., Antonopoulos N., Kanatas P., Karavas N., Bertoncelj I., Travlos I. (2022). Invasive alien plant species—Raising awareness of a threat to biodiversity and ecological connectivity (EC) in the Adriatic–Ionian region. Diversity.

[28] Yeo J.-K., Woo K.-S., Koo Y.-B., Kim Y.-S. (2007). Growth Performance and Adaptability of Three-Year-Old Poplar and Willow Clones in a Riparian Area. J. Korean Environ. Res. Reveg. Technol..

[29] Ashworth J., Keyes D., Kirk R., Lessard R. (2001). Standard Procedure in the Hydrometer Method for Particle Size Analysis. Commun. Soil Sci. Plant Anal..

[30] Thunjai T., Boyd C.E., Dube K. (2001). Poind Soil pH Measurement. J. World Aquac. Soc..

[31] Matejovic I. (1995). Total Nitrogen in Plant Material Determinated by Means of Dry Combustion: A Possible Alternative to Determination by Kjeldahl Digestion. Commun. Soil Sci. Plant Anal..

[32] Cox M.S. (2001). The Lancaster Soil Test Method as an Alternative to the Mehlich 3 Soil Test Method. Soil Sci..

[33] Aprile F., Lorandi R. (2012). Evaluation of Cation Exchange Capacity (CEC) in Tropical Soils Using Four Different Analytical Methods. J. Agric. Sci..

[34] David D.J. (1960). The Determination of Exchangeable Sodium, Potassium, Calcium and Magnesium in Soils by Atomic-Absorption Spectrophotometry. Analyst.

[35] R Core Team (2025). R: A Language and Environment for Statistical Computing.

[36] Zalesny R.S., Riemenschneider D.E., Hall R.B. (2005). Early rooting of dormant hardwood cuttings of *Populus*: Analysis of quantitative genetics and genotype × environment interactions. Can. J. For. Res..

[37] Sixto H., Salvia J., Barrio M., Ciria M.P., Cañellas I. (2011). Genetic variation and genotype-environment interactions in short rotation *Populus* plantations in southern Europe. New For..

[38] Pliura A., Lygis V., Suchockas V., Bartkevicius E. (2011). Performance of twenty four European Fraxinus excelsior populations in three Lithuanian progeny trials with a special emphasis on resistance to *Chalara fraxinea*. Baltic For..

[39] Mathew I., Shimelis H. (2022). Genetic analyses of root traits: Implications for environmental adaptation and new variety development: A review. Plant Breed..

[40] Schuster A., Santana A.S., Uberti A., Dias F.D.S., Dos Reis H.M., Destro V., DeLima R.O. (2024). Genetic diversity, relationships among traits and selection of tropical maize inbred lines for low-P tolerance based on root and shoot traits at seedling stage. Front. Plant Sci..

[41] Tian Y., Liu Y., Fang S., Yue J., Xu X. (2021). Genotypic Variations in 107 Poplar Clones Grown on a Short-Term Waterlogging Site: Long-Term (1992–2015) Data on Survival Rate, Growth Performance and Branching Traits. Data Brief.

[42] Lyon J., Gross N.M. (2005). Patterns of plant diversity and plant–environmental relationships across three riparian corridors. For. Ecol. Manag..

[43] Kinnoumè S.M.D., Gouwakinnou G.N., Noulèkoun F., Balagueman R.O., Houehanou T.D., Natta A.K. (2024). Trees diversity explains variations in biodiversity–ecosystem function relationships across environmental gradients and conservation status in riparian corridors. Front. For. Glob. Change.

